# 遗传性椭圆形红细胞增多症患者临床及基因突变特征：9例报告及文献复习

**DOI:** 10.3760/cma.j.issn.0253-2727.2023.04.009

**Published:** 2023-04

**Authors:** 旭 刘, 园 李, 馨 赵, 洋 杨, 莉 张, 丽萍 井, 蕾 叶, 康 周, 建平 李, 广新 彭, 慧慧 樊, 文睿 杨, 佑祯 熊, 凤奎 张

**Affiliations:** 中国医学科学院血液病医院（中国医学科学院血液学研究所），实验血液学国家重点实验室，国家血液系统疾病临床医学研究中心，细胞生态海河实验室，天津 300020 State Key Laboratory of Experimental Hematology, National Clinical Research Center for Blood Diseases, Haihe Laboratory of Cell Ecosystem, Institute of Hematology & Blood Diseases Hospital, Chinese Academy of Medical Sciences & Peking Union Medical College, Tianjin 300020, China

**Keywords:** 遗传性椭圆形红细胞增多症, 二代测序, 红细胞膜蛋白, 基因突变, SPTA1, Hereditary elliptocytosis, Next-generation sequencing, Erythrocyte membrane protein, Gene mutations, SPTA1

## Abstract

**目的:**

报告9例遗传性椭圆形红细胞增多症（hereditary elliptocytosis，HE）患者基因突变类型并分析HE致病性基因突变的特征。

**方法:**

报告中国医学科学院血液病医院2018年6月至2022年2月临床诊断的9例HE患者临床及基因突变特征，应用Sanger测序方法进行验证，分析基因突变构成、突变类型、基因型及与临床表型之间的关系。

**结果:**

9例HE患者中6例为SPTA1、1例为SPTB、1例为EPB41红细胞膜蛋白基因突变，另1例为20号染色体拷贝缺失。共涉及11个基因突变位点，包括6个已知突变和5个新发突变。5个新发突变分别为：SPTA1基因9号外显子c.1247A>C（p.K416T），15号外显子c.1891delG（p.A631fs*17），6～12号外显子Del；SPTB基因c.154C>T（p.R52W）；EPB41基因c.1636A>G（p.I546V）。6例SPTA1突变患者中3例为SPTA1 9号外显子突变。

**结论:**

SPTA1是HE患者最常见的突变基因。

## Abstract (en)

**Objective:**

To report gene mutations in nine patients with hereditary elliptocytosis（HE）and analyze the characteristics of pathogenic gene mutations in HE.

**Methods:**

The clinical and gene mutations of nine patients clinically diagnosed with HE at Institute of Hematology & Blood Diseases Hospital from June 2018 to February 2022 were reported and verified by next-generation sequencing to analyze the relationship between gene mutations and clinical phenotypes.

**Results:**

Erythrocyte membrane protein gene mutations were detected among nine patients with HE, including six with SPTA1 mutation, one with SPTB mutation, one with EPB41 mutation, and one with chromosome 20 copy deletion. A total of 11 gene mutation sites were involved, including 6 known mutations and 5 novel mutations. The five novel mutations included SPTA1: c.1247A>C（p. K416T）in exon 9, c.1891delG（p. A631fs*17）in exon 15, E6-E12 Del; SPTB: c.154C>T（p. R52W）; and EPB41: c.1636A>G（p. I546V）. Three of the six patients with the SPTA1 mutation were SPTA1 exon 9 mutation.

**Conclusion:**

SPTA1 is the most common mutant gene in patients with HE.

遗传性椭圆形红细胞增多症（hereditary elliptocytosis，HE）是以外周血椭圆形红细胞增多为特征的遗传性溶血性疾病。由编码红细胞膜或骨架蛋白的基因突变引起红细胞膜异常所致，主要呈常染色体显性遗传，主要涉及SPTA1、SPTB和EPB41基因，分别编码ɑ血影蛋白、β血影蛋白和带4.1蛋白[1]。HE在世界各地均有分布，在欧美国家患病率为0.03％～0.05％[2]，在非洲等疟疾流行地区患病率可达0.6％～1.6％[3]。文献中HE多为散在的个例或家系报道，多数HE无明显临床症状，容易与遗传性球形红细胞增多症混淆。了解HE的基因突变特点，以及基因型与临床表型的关系对于HE的精准诊断具有重要的意义。我们报告本中心确诊的9例HE患者，总结HE基因突变特点如下。

## 病例与方法

1. 病例：本研究为回顾性研究，以2018年6月至2022年2月中国医学科学院血液病医院贫血诊疗中心连续收治的8个独立家系的9例HE患者为研究对象。患者经详细的病史采集、体格检查、实验室和影像学检查，并应用二代基因测序（NGS）确定基因型，依照文献[4]标准明确诊断为HE。

2. 实验室和影像学检查：血常规，外周血涂片，游离血红蛋白（F-HB），结合珠蛋白（HP），成熟红细胞寿命测定（内源性CO呼气试验），红细胞膜病相关溶血试验［红细胞渗透脆性试验（EOF）、酸化甘油溶血试验（AGLT50）、伊红-5′-马来酰亚胺（EMA）检测］，红细胞酶病相关溶血试验［葡萄糖-6-磷酸脱氢酶（G6PD）、丙酮酸激酶（PK）、红细胞嘧啶5′-核苷酸酶（P5′N）］，血红蛋白病相关溶血试验［血红蛋白A2（HbA2）、血红蛋白F（HbF）］，获得性溶血性贫血试验［直接抗球蛋白试验（Coombs）、酸溶血试验、冷凝集素试验］，消化系统超声检查等。

3. 基因突变测序：参照文献[5]方法进行NGS检测，平均测序覆盖度为99.59％，平均测序深度569.67×，将测序结果与人类基因突变数据库（HGMD）（http://www.hgmd.cf.ac.uk/ac/index.php）及千人基因组数据库进行比对。根据美国医学遗传学与基因组学会（ACMG）发布的变异解读指南进行致病性分析，并结合患者的临床特点和家族史，对患者及其父母的DNA采用了Sanger测序法进行突变位点验证，从而确定患者的致病性突变。

## 结果

1. 一般情况与临床特征：9例患者中，男3例，女6例，中位年龄40（26，55）岁，中位确诊年龄为32（21，40）岁，中位病史8（0.5，20）年。8例脾脏肿大，5例皮肤及巩膜黄染，4例明显乏力，3例尿色加深，2例间断发热。有贫血相关家族史者6例。

2. 血液学参数及溶血检验：9例患者中有8例存在轻中度贫血，中位HGB 84（74，93）g/L；中位WBC 5.31（2.81，5.44）×10^9^/L，中位PLT 184（147，238）×10^9^/L，中位网织红细胞比例（RET％）10.69％（7.85％，14.56％），中位网织红细胞绝对值（ARC）0.236（0.182，0.379）×10^9^/L，中位平均红细胞体积（MCV）104.4（97.0，114.9）fl，中位平均血红蛋白浓度（MCH）34.7（32.5，36.8）pg，中位平均红细胞血红蛋白浓度（MCHC）333（320，342）g/L，中位红细胞体积分布宽度（RDW-SD）84.75（79.73，87.38）fl。7例检验了血清LDH的患者中5例高于正常值（>247 U/L）。5例患者的F-HB高于正常值上限（40 mg/L），6例患者HP低于正常值下限（0.5 g/L）。4例患者（例2、例5、例7、例8b）检测了红细胞寿命，分别为9、24、13、14 d。7例患者AGLT50试验阳性（<290 s）。在8例进行了EOF试验的患者中7例显示开始溶血时间延长，红细胞渗透脆性增加。6例患者EMA试验均正常。

3. 外周血红细胞形态学检查及骨髓细胞学检查：外周血涂片可见成熟红细胞大小不一，椭圆形红细胞占50％～90％，偶见球形红细胞和红细胞碎片。骨髓涂片有核细胞增生明显活跃，红系比例明显增高（35.3％～77.5％）。

4. 红细胞膜蛋白基因突变：9例患者检出红细胞膜蛋白突变基因分别涉及SPTA1、SPTB和EPB41，共检出11个基因突变位点。其中SPTA1基因突变6例（分别为单等位基因杂合突变3例、双位点复合杂合突变2例、6～12号外显子片段缺失1例），SPTB突变1例，EPB41突变1例，20号染色体拷贝缺失1例。11个突变的基因位点包含9个基因点突变和2个基因大片段缺失，其中5个为新发突变，6个为已知突变。5个新发突变分别为：SPTA1基因的9号外显子c.1247A>C（p.K416T）；15号外显子c.1891delG（p.A631fs*17）为致病性突变；6～12号外显子Del为可疑致病性；SPTB基因 c.154C>T（p.R52W）和EPB41基因c.1636A>G（p.I546V），致病性分析均为未确定。6个已知突变中有5个来自SPTA1基因，另一个为20号染色体拷贝缺失（表1）。

**表1:** 9例遗传性椭圆形红细胞增多症（HE）患者的基因突变情况

患者	诊断	基因	外显子	转录本	核苷酸改变	氨基酸改变	突变类型	状态	致病性分析
例1	HE	SPTB	2	NM_001024858	c.154C>T	p.R52W	错义突变	新发	未确定
例2	HE	SPTA1	6～12	NM_003126.2	Del	–	–	新发	可疑致病性
例3	HE	SPTA1	2	NM_003126	c.83G>A	p.R28H	错义突变	已知	可疑致病性
		SPTA1	9	NM_003126	c.1247A>C	p.K416T	错义突变	新发	未确定
例4	HE	SPTA1	15	NM_003126	c.1891delG	p.A631fs*17	移码突变	新发	致病性
例5	HE	EPB41	17	NM_004437	c.1636A>G	p.I546V	错义突变	新发	未确定
例6	HE	20号拷贝缺失染色体	–	20q11.22q13.13	–	–	–	已知	–
例7	HE	SPTA1	38	NM_003126	c.5410C>T	p.L1804F	错义突变	已知	未确定
例8a^†^	HE	SPTA1	9	NM_003126	c.1120C>T	p.R374X	无义突变	已知	致病性
		SPTA1	44	NM_003126	c.6235C>T	p.R2079W	错义突变	已知	未确定
例8b^†^	HE	SPTA1	9	NM_003126.2	c.1120C>T	p.R374X	无义突变	已知	致病性

**注**：–：不适用；†：例8a和例8b来自同一家系，例8b为例8a之女

3例患者（例3、例8a、例8b）携带SPTA1基因9号外显子突变，贫血程度各不相同。例3、例8a均有SPTA1基因的复合杂合突变，例3呈中度贫血，例8a外周血涂片中可见椭圆形红细胞且伴有脾大症状，但无贫血。例8b与例8a来自同一家系，为例8a之女，仅遗传了父亲的SPTA1突变c.1120C>T（p.R374X），呈中度贫血。

有3例患者（例1、例3、例5）进行了父母亲代基因突变位点的Sanger测序验证。其中例1 SPTB基因2号外显子的突变c.154C>T（p.R52W）经验证源自父亲。例3 SPTA1基因复合杂合突变，c.83G>A（p.R28H）源自父亲，c.1247A>C（p.K416T）源自母亲。例5 EPB41基因17号外显子突变c.1636A>G（p.I546V）遗传自母亲。

## 讨论

HE常见突变的红细胞膜蛋白基因涉及SPTA1、EBP41和SPTB。SPTA1是最常突变的基因，且突变主要发生在2号外显子，以点突变为主，错义突变是最主要的突变类型。HE的基因型和临床表型间似无明显相关性。我们推测SPTA1基因2号外显子上的c.82-c.83区域突变所致的28号氨基酸改变，40号外显子的c.5572C>G（p.Leu1858Val）可能为HE患者SPTA1基因的高频突变。

为了探究HE基因型与临床表型之间的潜在关联，我们汇总了Pubmed（检索词：hereditary elliptocytosis）2000至2022年国内外进行了基因检测的HE病例并对其进行分析。来自不同家系的30例HE患者（文献报道22例，本研究8例）共涉及36个膜蛋白基因位点（附表）。其中，SPTA1（27/36，75.0％）是HE患者最常见的突变基因，其次分别是EPB41（7/36，19.4％）和SPTB（2/36，5.6％）。27例SPTA1突变的基因位点中，24例为点突变，且SPTA1突变主要发生在2号外显子（8/24，33.3％），同时也在其他外显子散在分布。在中国患者的14个SPTA1基因突变位点中，突变仍主要发生于2号外显子（4/14，28.6％）。错义突变（18/24，75％）是SPTA1最主要的点突变类型。无义突变（3/6，50％）是EPB41基因最主要的点突变类型。

综合文献报道与本研究病例，SPTA1基因2号外显子发生突变的8例HE患者贫血程度各不相同，这或许与突变位点的不完全外显有关，其中一个基因很可能发生沉默并未表达。Motulsky等[6]也曾有类似推测，可通过检测相应mRNA表达进一步验证。来自不同家系的3例HE患者均为SPTA1基因c.82C>T（p.R28C）突变[7]–[9]，分别呈轻/中度贫血或不贫血。同一家系中有SPTA1基因c.1120C>T（p.R374X）致病突变的父亲（例8a）无贫血，女儿（例8b）呈中度贫血。由于不同个体间SPTA1基因编码α链数量是可变的，因此相同基因突变的不同个体间临床表现不尽相同。

SPTA1基因位于1q23.1上，包含52个外显子，其所编码的α血影蛋白是红细胞骨架蛋白的主要成分，该基因突变时将影响红细胞骨架的水平联结，导致红细胞的稳定性和变形能力下降发生溶血[10]。既往研究发现，α血影蛋白NH2末端区域的错义突变是HE和遗传性热异形红细胞增多症（HPP）中最常见的缺陷之一[11]。28号密码子突变所致氨基酸Arg28改变是非洲裔美国人SPTA1基因的热点突变[12]。依据本次文献汇总结果，3例不同家系患者中均发现了SPTA1基因2号外显子错义突变c.82C>T（p.R28C）[7]–[9]；3例不同家系患者SPTA1基因的2号外显子错义突变c.83G>A（p.R28H）[7],[13]。故推测28号氨基酸在α血影蛋白的结构和功能中发挥着重要的作用。在两个不同家系患者中发现SPTA1基因的40号外显子错义突变c.5572C>G（p.Leu1858Val）[7],[14]。因此我们推测2号外显子上的c.82-c.83区域突变所致的28号氨基酸改变，40号外显子的c.5572C>G（p.Leu1858Val）可能为HE患者SPTA1基因的高频突变。

EBP41基因位于1p35.3上，包含28个外显子，编码4.1R蛋白，该基因突变所致的4.1R蛋白缺陷会影响细胞膜骨架完整性从而发生溶血。4.1R的部分缺乏可见于白种人HE患者，表现为轻度HE，很少或几乎没有溶血[15]–[18]。4.1R蛋白完全缺失患者会发生严重溶血[19]–[22]。故4.1R蛋白缺陷程度与患者的临床表型密切相关，单一杂合EPB41基因突变的个体有轻/中度贫血，而EPB41基因纯合突变所致4.1R蛋白完全缺失患者表现为重度贫血。

SPTB基因位于14q23.3上，包含38个外显子，编码的β血影蛋白是细胞膜骨架的重要组成部分。β血影蛋白COOH末端区域的截断突变（包括插入、缺失、无义突变等）会影响ɑ-β二聚体重复区进而影响四聚体结构的形成，这是HE或HPP常见致病缺陷[11],[23]。结合文献报告发生SPTB突变的HE患者仅有2例：c.6203 T>C（p.L2068P）[24]和c.154C>T（p.R52W）均为错义突变，均为中度贫血。因病例数较少，故SPTB基因与HE临床表型之间关系仍是未知。

我们的研究也存在不足之处，由于大部分HE患者没有临床症状不易发现，因而目前报道的完善基因检查的样本例数较少，HE的基因型与表现型之间的对应关系尚未阐明。与例6相似，既往曾报道伴有染色体20q−的MDS患者外周血涂片发现椭圆形红细胞增多但原因尚不明[25]–[27]。

NGS技术对于遗传性疾病的诊断提供了有力支持，相信随着NGS的广泛应用和病例数的累积，HE基因型与表现型之间的关系将更加明晰，从而实现遗传性疾病的分子诊断新时代。
